# Are Technology-Driven Mobile Phone Applications (Apps) the New Currency for Digital Stent Registries and Patient Communication: Prospective Outcomes Using Urostentz App

**DOI:** 10.1155/2021/6612371

**Published:** 2021-01-06

**Authors:** B. M. Zeeshan Hameed, Milap J. Shah, Nithesh Naik, Mohan Amaresh, Padmaraj Hegde, Rahil Hussein Beary, Suraj Jayadeva, Bhaskar K. Somani

**Affiliations:** ^1^Department of Urology, Kasturba Medical College and Hospital, Manipal Academy of Higher Education, Manipal, India; ^2^Chief of Innovation Centre, Kasturba Medical College, Manipal Academy of Higher Education, Manipal, India; ^3^Faculty of Engineering, Manipal Institute of Technology, Manipal Academy of Higher Education, Manipal, India; ^4^Kasturba Medical College, Manipal Academy of Higher Education, Manipal, India; ^5^Department of Urology, University Hospital Southampton NHS Trust, Southampton, UK

## Abstract

**Background:**

Forgotten ureteral stents (FUS) and stent-related symptoms (SRS) lead to increased postprocedural emergency department visits and add to the psychological and financial burden of the patients.

**Purpose:**

To review the usage and benefits of ureteral stent tracking and symptom monitoring through a single smartphone-based application (App) platform with 2-way clinician-patient communication. This study also compared the features with other smartphone apps used for stent tracking.

**Materials and Methods:**

100 patients were included in this single-center prospective study conducted between September 2019 and December 2019. Patients who had metallic or long-term indwelling stents, noncomprehensible patients, and those not willing to share their data were excluded from the study.

**Results:**

Of 100 patients, 92 downloaded the Urostentz application, and 72 (78.2%) patients answered the pictorial symptom questionnaire. Symptom score analysis suggested that 62 patients (86.1%) had stent-related symptoms of which 3 required readmission and underwent early stent removal. The mean stent duration was 17.2 + 3.5 days (range: 11–23 days), with 69% of patients having their stent removed on the scheduled date and 25% of patients requesting a change of their appointment via the App.

**Conclusion:**

In this study, there was no case of FUS encountered. The “Urostentz” App is a freely available patient safety stent tracking application that provides a secure and simplified interface, which can significantly reduce the incidence of FUS and provide digital remote assistance in the management of stent-related symptoms.

## 1. Introduction

Ureteral stents are a vital part of the present-day endourological practice. The indications for ureteral stent insertion consist of operative interventions involving the kidney or ureter as well any condition causing internal or external obstruction of the ureter [1–4]. According to the literature, 12% of all stents remain indwelling beyond their maximal safe life [2]. Such stents are termed as forgotten ureteral stent (FUS). FUS can be associated with complications such as migration, infection, encrustation, stent fracture, renal failure, and sepsis [3]. More than 75% of stents are found to be encrusted when removed after 12 weeks of insertion [2, 3]. Stent-related symptoms (SRS) such as dysuria, loin pain, haematuria, urinary incontinence, and lower urinary tract symptoms (LUTS) are observed in nearly 80% of stented cases [4]. The removal of FUS also posed a challenge to urologists. Multiple endourological procedures may be required for the removal of infected, fractured, or encrusted FUS. In addition to the increased costs of postprocedure-related events (PRE) such as multiple clinic appointments, readmissions, and emergency department visits, there is also an added burden of medicolegal issues associated with it. This adds to an increased physical, psychological, and economic burden on the patients [4, 5].

One of the important components of using ureteral stents is creating an effective structure to track and monitor these stents and ensure their timely removal. At present, there is no standard universally accepted protocol regarding their follow-up and retrieval. Despite the existence of few stent registries, mishaps continue to happen and there are significant patient and financial benefits to be sought from engendering the use of a robust system. Few stent tracking applications are available but none of them addresses the issues related to the SRS or allows 2-way communication with the patients. Hence, we developed a smartphone application that helps in tracking the ureteral “stents” as well as monitoring the “symptoms” associated with these stents via a 2-way clinician-patient communication.

In this study, we aimed to evaluate the usefulness of the “Urostentz” application in ureteral stent tracking, SRS monitoring, and compare the features with other smartphone applications used for stent tracking.

## 2. Materials and Methods

### 2.1. Patients

This was a single-center study conducted in our university teaching hospital. Institutional Ethics Committee clearance and certificate was obtained for the study (IEC: 651/2019). Over 4 months between September 2019 and December 2019, prospective outcomes were recorded for consecutive patients in whom ureteral stenting was done for any urological indication. All procedures performed in studies involving human participants were in accordance with the ethical standards of the institutional and/or national research committee and with the 1964 Helsinki declaration and its later amendments or comparable ethical standards.

The inclusion criteria were all patients having ureteral stent insertion (polyurethane) and patients or their immediate relatives having a smartphone or analog phone willing to participate in the study. Patients who had metallic or long term indwelling stents, noncomprehensible patients, and those not willing to share their data were excluded from the study. The total number of patient recruitment was limited to the first 100 patients for this pilot study.

### 2.2. Mobile Phone Application (App)

In 2019, “Urostentz” was developed to streamline the tracking of ureteral stents and stent-related symptoms, improve patient safety by creating an effective medium of communication, and improve the quality of data collection. Urostentz is one of its kind smartphone applications, which also provides the facility to track the SRS of the patients and provide digital remote assistance for the same. It is freely available to download on Google Playstore. It can also be accessed on the computer by visiting the website http://www.urostentz.com. The iOS application is submitted and the same shall be available soon on the App store.

#### 2.2.1. Protocol

After double J stent insertion for urological procedures, the application was downloaded in the patient's or relative's smartphone, linked to a profile that was created under the treating surgeon, containing the details related to the procedure and the stent insertion.

**Figure 1:**
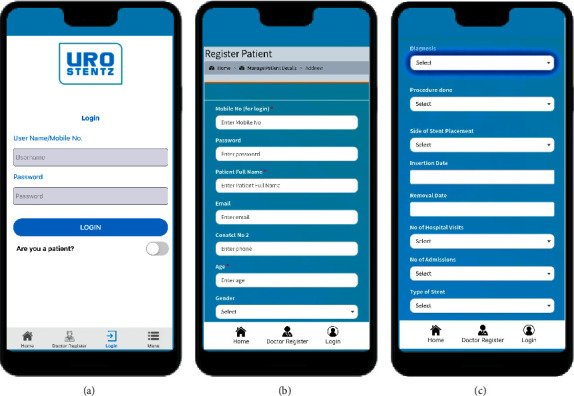
(a) Doctor and patient login. (b) and (c) Patient registration details including demographics, indication for stent insertion, stent size, length, date of its insertion, and scheduled removal date (reproduced with the permission of developers of the application (RAYZ CHARTERED)).

All patients were registered under the experienced treating surgical team. Once the patient was registered, the details were protected as only the treating surgeon could access the data. All information obtained was encrypted and kept secure. In each case, details such as patient demographics, indication for stent insertion, stent size, length, date of its insertion, and scheduled removal date were entered as shown in Figures 1(a)–1(c). This information was stored in the database which could be accessed for audit and gave the entire information with search options as per the requirement.

**Figure 2:**
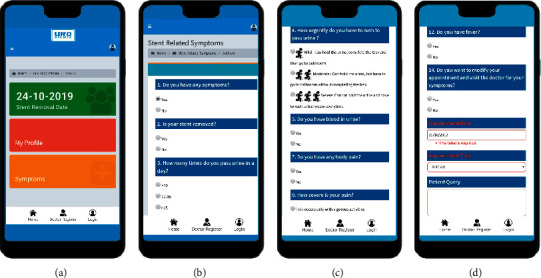
(a) Patient dashboard showing the scheduled date of stent removal, access to symptom questionnaire, and personal profile. (b–d) Pictorial symptom questionnaire for patients with stent-related symptoms and symptoms even after stent removal (reproduced with the permission of developers of the application (RAYZ CHARTERED)).

The patient could log in from their smartphone or web browser and access information regarding the scheduled date of stent removal (Figure 2(a)). In the case of SRS, the patient could also access the pictorial questionnaire and submit details regarding their symptoms to the clinician. The decision to include the stent-related symptoms was derived using a “Delphi” method involving six experienced endourologists.

This questionnaire was based on the pictorial depiction of the symptoms from the Ureteral Stent Symptom Questionnaire (USSQ) to improve patient compliance (Figures 2(b)–2(d)) [6]. The application also provided the patient with the facility to request a change in the scheduled appointment.

**Figure 3:**
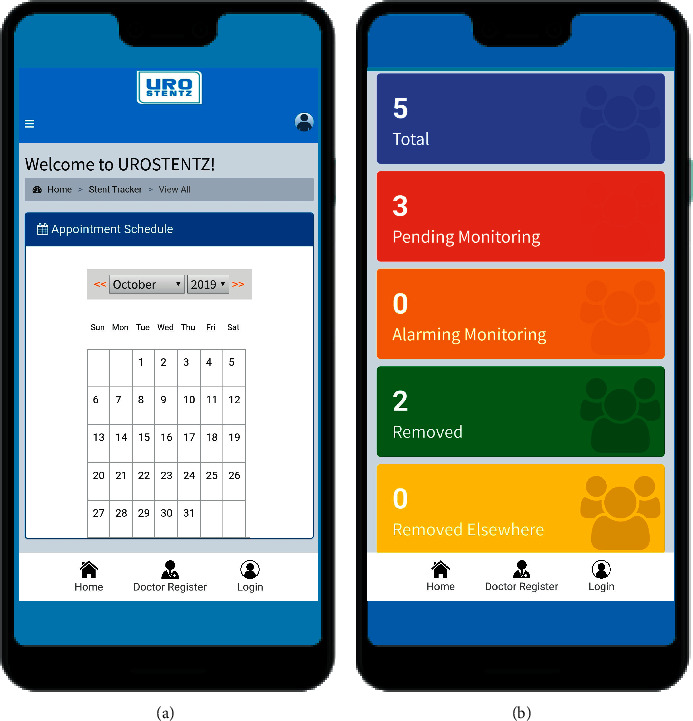
(a) Clinician dashboard to receive notifications regarding the total patients scheduled for stent removal on a day-to-day basis. (b) Clinician dashboard: updates regarding patient's ureteral stent removal status (reproduced with the permission of developers of the application (RAYZ CHARTERED)).

The clinician received notifications regarding the patient queries and responded with a message accordingly. The patient received reminders regarding the stent removal date as scheduled by the clinician. The clinician was also notified regarding the total patients scheduled for stent removal on a day-to-day basis to plan their stent removals (Figure 3(a)). When a patient reported for stent removal, the entry was updated as “removed” in the stent dashboard (Figure 3(b)).

A patient who did not return for stent removal within 3 months of their scheduled date was categorized as FUS. A patient could only have 1 profile but could have more than 1 ureteral stent care plan during the study period. Bilateral stent placement done in the same sitting was considered as a single stent care plan. As per standard protocol, all stents were removed after 2-3 weeks from the date of insertion after confirming the stone-free status with X-ray or ultrasound for radioopaque stones and NCCT (noncontrast computerized tomography) scan of the KUB (kidneys ureter bladder) for radiolucent stones.

#### 2.2.2. Outcome Measures

The primary outcome measures were the incidence of FUS and stent-related symptom analysis based on the questionnaire answered. The secondary outcome measures were adherence to the scheduled date of removal, rate of outpatient visits, and readmissions. The number of patient communication episodes was also captured.

### 2.3. Statistical Analysis

Data extracted included patient demographics, diagnosis, procedure, stent characteristics, use of smartphones or web browsers, stent-related questionnaire response, and adherence to the scheduled appointment. Descriptive statistics were performed to calculate mean, standard deviation, and range using IBM SPSS Statistics for Windows, Version 24 (IBM Corp., Armonk, NY).

## 3. Results

An initial group of 100 patients underwent ureteral stent placement for various indications and were subsequently tracked using the “Urostentz” application. The details regarding patient demographics, symptom score analysis, and adherence to the schedule are described in Table 1.

The mean patient age was 42.3 ± 13.7 years (range: 14–80 years), with a male : female ratio of 2 : 1. Stent placement was done after percutaneous nephrolithotomy (PCNL) (*n* = 44), after ureterocopy (URS) (*n* = 48), and other procedures (*n* = 8). Of 100 patients, 92 had smartphone access and were able to download the Urostentz application while the other 8 patients were registered through the website. 72 (78.2%) patients answered the pictorial symptom questionnaire through the Urostentz application. Based on the answers, the symptom score analysis suggested that 10 patients (13.8%) had no symptoms and 62 patients (86.1%) had stent-related symptoms. Three (3%) patients required readmission due to severe loin pain (*n* = 2) and pyelonephritis (*n* = 1). All 3 patients underwent early stent removal. 15 (15%) patients were managed conservatively for their symptoms on an outpatient department (OPD) basis. 69% of patients had their stent removed on the scheduled date while 25% of patients requested a change in appointment. Overall, 6 (6%) cases had delayed stent removal. The mean stent duration was 17.25 ± 3.5 days (range: 11–23 days). There was no case of FUS encountered in the study.

## 4. Discussion

To our knowledge, this is the first study on ureteral stent tracking and symptom monitoring through a single smartphone-based app platform. The preliminary data from this pilot study suggest that the Urostentz application is an effective mode to track the patients with indwelling ureteral stents and monitor their stent-related symptoms.

Smartphones have become an integral part of our routine life. Various smartphone-based applications have been developed for tracking patients with indwelling ureteral stents. In 2016, Boston Scientific in partnership with Visible Health launched the Ureteral Stent Tracker (UST) application. Other smartphone-based applications launched in 2019 were Stent Tracker, Stone MD, and Double J Tracker. The authors have discussed the features of the different available applications and their limitations and given a comprehensive comparison of the same in Table 2. Urostentz app is the only smartphone application with stent tracking as well as stent-related symptom tracking. This is a unique application that helps in two-way objective communication between patients and the urologist.

Various studies have been conducted using the UST application to check its clinical application. In 2017, Molina et al. reported their retrospective study using the UST. In this study, 77% of stents were removed on time while 9% were overdue. Only 1 out of 194 patients were lost to follow-up [7]. Ziemba et al. (2017) reported that 3 out of 115 patients (3%) who did not return for their scheduled stent removal could be identified only through the UST application [8]. Ulker et al. [9] used UST and compared it with the appointment card registry to track patients with indwelling ureteric stents. The results showed that patients could easily be recalled through UST [9]. Similar to these studies, in our series using the Urostentz application there was no incidence of FUS.

In literature, SRS include storage and voiding symptoms such as urgency (57–60%), frequency (50–60%), incomplete emptying (76%), dysuria (40%), suprapubic pain (30%), flank pain (19–32%), haematuria (25%), and incontinence [10–17]. Stent placement is also associated with increased postprocedure-related events (PREs) including phone calls, extraclinic appointments, and emergency department visits [18]. This adds to increased psychological as well as an economic burden on the patients. The clinicians also have the burden to attend to these extra calls and patient visits. There is no system or software designed to help reduce these stent-related issues. Urostentz is the first smartphone-based application that addresses this issue where the App is applied clinically to track and monitor SRS. We monitored the SRS of patients who answered the pictorial questionnaire through the App. Out of 72 patients who answered the questionnaire, only 18 patients had to revisit the hospital for the same while the rest of the patients were reassured and none of them reported any further complaints or complications.

Although our sample size was small and based on a single institution, it is a pilot study and the objective was to check the efficacy and usefulness of Urostentz application. The authors plan to further evaluate the effectiveness of this application in reducing the incidence of FUS and SRS by utilizing this application in a larger cohort through a multi-institutional global study. The impact of this application in reducing the economic burden on patients having stent-related symptoms also needs to be evaluated. Since the Urostentz app is currently available only on Google Playstore, the wider application of this App after its availability on iOS phones needs to be evaluated.

## 5. Conclusion

The “Urostentz” App is a freely available patient safety stent tracking application that provides a secure and simplified interface, which can significantly reduce the incidence of FUS and provide digital remote assistance in the management of stent-related symptoms. In case of patients who have passed their scheduled date of stent removal, this application can provide an effective medium for 2-way clinician-patient communication to notify the patients and also avoid unnecessary hospital visits in case of insignificant SRS thereby reducing the financial burden on the patients.

## Figures and Tables

**Table 1:** Patient demographics, symptom score analysis, and adherence to the schedule.

*Patient demographics (N = 100)*	
Age (mean ± SD) in years	42.3 ± 13.76 (range: 14–80)
Male : female	2 : 1
Right: left: bilateral	52 (52%): 40 (40%): 8 (8%)
PCNL : URS : others	44 (44%): 48 (48%): 8 (8%)
Smartphones: website	92 (92%): 8 (8%)
*Symptom score analysis*	
No symptoms	10 (13.8%)
Symptoms present	62 (86.1%)
Readmissions	03 (3%)
OPD visits only	15 (15%)
*Adherence to the stent removal schedule*	
Mean (+/−SD) stent duration (days)	17.25 ± 3.54 (range: 11–23)
Stent removal on the scheduled date	69
Delayed stent removal (*n* = 6): ≤3 days, 3–7 days	4 patients, 2 patients
Stent postponed as per the patient request (via app)	25 patients
Forgotten ureteral stent (SUF)	Nil

**Table 2:** Comparison of smartphone applications for ureteral stent tracking.

Features	Urostentz	Ureteral Stent Tracker ^*∗*^ by Visible Health	Stent Tracker	Double J Tracker	Stone MD
Platforms available					
Android	+	+	+	+	+
iOS	Submitted	+	+	—	+
Web browser	+	+	—	—	—
Access	Free	Withdrawn (was only accessible to registered doctors)	Free	Paid 14 days of a free trial Rs 7900/year	Free
Languages	English	English	English	English	English
Doctors dashboard	Yes	Yes	Yes	NA	No
Patient dashboard	Yes	No	No	NA	Yes
Patient education dashboard	Yes	No	No	NA	Yes
Symptom tracking	Yes	No	No	NA	No
Two-way communication between the doctor and the patient	Present	Absent	Absent	Absent	Absent
Personalized message facility	Yes	No	No	NA	No
Notifications/reminders	Yes	Yes	Yes	NA	Self-reminder
Medium of notifications	Short messaging service (SMS) E-mail (if provided)	E-mail	NA	NA	NA
Timing of reminders	1 week before the scheduled date On the day of the scheduled date 1 day after the scheduled date (if the patient does not follow-up)	Daily or weekly as per requirement	NA	NA	On the day of the scheduled date
Facility for change in appointment	Yes	No	No	NA	NA
Record of total hospital visits/consultations/readmissions	Yes	No	No	NA	NA
Limitations	Not integrated to institutional EMR	Limited to physicians who are registered and preauthorized by Boston Scientific Not integrated to institutional EMR No monitoring of SRS Withdrawn by the company	Single language No SRS monitoring facility Not available on the web browser No automated reminders or messages for stent removal Limited to the users defined by the pharma company	Single language No SRS monitoring facility Not available on the web browser Only available on iOS Paid services	Single language No reminders to the doctors No two-way communication for stent removal No SRS monitoring facility Not available on the web browser

^*∗*^The app was withdrawn from both iOS and Android platforms.

## Data Availability

The data were collected for carrying out this work from Urostentz mobile application registry.

## Abbreviations

FUS: Forgotten ureteral stents
SRS: Stent-related symptoms
LUTS: Lower urinary tract symptoms
PRE: Postprocedure-related events
USSQ: Ureteral Stent Symptom Questionnaire
PCNL: Percutaneous nephrolithotomy
OPD: Outpatient department
UST: Ureteral Stent Tracker
NCCT: Noncontrast computerized tomography
KUB: Kidneys ureter bladder.
